# Investigating the Influence of Fe Speciation on N_2_O Decomposition Over Fe–ZSM-5 Catalysts

**DOI:** 10.1007/s11244-018-1024-0

**Published:** 2018-07-24

**Authors:** Nia Richards, Ewa Nowicka, James H. Carter, David J. Morgan, Nicholas F. Dummer, Stanislaw Golunski, Graham J. Hutchings

**Affiliations:** Cardiff Catalysis Institute, School of Chemistry, Cardiff University, Cardiff, CF10 3AT UK

**Keywords:** Nitrous oxide, Iron zeolites, Fe–ZSM-5, N_2_O decomposition, Acid washing, Iron species, Chemical vapour impregnation, UV/Vis, XPS

## Abstract

The influence of Fe speciation on the decomposition rates of N_2_O over Fe–ZSM-5 catalysts prepared by Chemical Vapour Impregnation were investigated. Various weight loadings of Fe–ZSM-5 catalysts were prepared from the parent zeolite H-ZSM-5 with a Si:Al ratio of 23 or 30. The effect of Si:Al ratio and Fe weight loading was initially investigated before focussing on a single weight loading and the effects of acid washing on catalyst activity and iron speciation. UV/Vis spectroscopy, surface area analysis, XPS and ICP-OES of the acid washed catalysts indicated a reduction of ca. 60% of Fe loading when compared to the parent catalyst with a 0.4 wt% Fe loading. The TOF of N_2_O decomposition at 600 °C improved to 3.99 × 10^3^ s^−1^ over the acid washed catalyst which had a weight loading of 0.16%, in contrast, the parent catalyst had a TOF of 1.60 × 10^3^ s^−1^. Propane was added to the gas stream to act as a reductant and remove any inhibiting oxygen species that remain on the surface of the catalyst. Comparison of catalysts with relatively high and low Fe loadings achieved comparable levels of N_2_O decomposition when propane is present. When only N_2_O is present, low metal loading Fe–ZSM-5 catalysts are not capable of achieving high conversions due to the low proximity of active framework Fe^3+^ ions and extra-framework ɑ-Fe species, which limits oxygen desorption. Acid washing extracts Fe from these active sites and deposits it on the surface of the catalyst as Fe_x_O_y_, leading to a drop in activity. The Fe species present in the catalyst were identified using UV/Vis spectroscopy and speculate on the active species. We consider high loadings of Fe do not lead to an active catalyst when propane is present due to the formation of Fe_x_O_y_ nanoparticles and clusters during catalyst preparation. These are inactive species which lead to a decrease in overall efficiency of the Fe ions and consequentially a lower TOF.

## Introduction

Nitrous oxide (N_2_O) is a highly potent greenhouse gas, having a global warming potential of roughly 300, therefore the effect on the atmosphere is far more devastating than carbon dioxide [1–3]. N_2_O is produced by both natural and anthropogenic sources [4–6]. There are many sources of anthropogenic N_2_O such as sewage treatment, fuel and biomass combustion, industrial chemical processes, and contributions from the agriculture sector [4]. Agricultural processes lead to around 60% of the global emissions. The main industrial processes that lead to the formation of N_2_O such as adipic and nitric acid production [1], with adipic acid production leading to around 80% of the global industrial emission of N_2_O (~ 10% total) [6, 7]. There are also small industrial uses such as hospital and dental surgeries [8]. Therefore it is extremely important to decompose N_2_O before it is released into the atmosphere. Decomposition of N_2_O takes place through dissociation into O_2_ and N_2_ (Eq. 1, [9, 10]).

1 $$2{{\text{N}}_2}{{\text{O}}}\rightarrow\;{{\text{N}}_2}+\;{{\text{O}}_2}\;({\Delta _{\text{r}}}{\text{H}} \circ \left( {298} \right)= - 163\;{\text{kJ}}\;{\text{mo}}{{\text{l}}^{ - 1}})$$

There are many types of catalysts that can be used for N_2_O decomposition including perovskites [11–15], ceria-based catalysts [16–18], spinels [19–21] and iron containing zeolites [22]. In the latter case, H-ZSM-5 has been frequently used as a support [23–25]. Xie et al. reported 100% conversion at 450 °C using 7.46 wt % Fe [26] while Wood and co-workers [27] reported 84% conversion at 500 °C using an Fe/ZSM-5 catalyst with a loading of 0.57 wt%. Sobalik et al. showed that when using Ferrierite (FER) a Si:Al ratio of 8.5 outperformed Si:Al 10.5 when the same Fe loading is prepared for N_2_O decomposition [28]. Rauscher et al. reported that low Si:Al ratios are more effective for N_2_O decomposition catalysts [23]. Fe–ZSM-5 (Si:Al = 11.4) exhibited 95% conversion of N_2_O at 500 °C in contrast to Fe-BEA (93) achieving just 20% conversion of N_2_O at 575 °C [29]. The work of both these groups show that Si/Al ratio is an important factor for activity of an N_2_O decomposition catalyst.

Furthermore, it was shown that zeolites with different framework structures can be used for the decomposition of N_2_O with MFI (ZSM-5), beta (BEA) and ferrierite (FER) zeolites acting as supports for Fe [29–31]. Jisa et al. reported that low loaded Fe-FER was the most active, achieving 85% conversion at 450 °C [32]. FER had the lowest Si/Al ratio (8.6) out of all the zeolites tested compared to 15.5 for BEA and 13.4 for MFI. Supporting the earlier findings that a low Si:Al ratio is necessary for high N_2_O conversion. As the active Fe site is considered to form on the Al moiety in the zeolite framework, low Si:Al ratio zeolites can facilitate a higher concentration of active species [26].

The rate-limiting step in the decomposition of N_2_O is typically the recombination of oxygen to form O_2_. Specifically, the dissociation of N_2_O on the active Fe species is facile and leaves an oxidised Fe active site. The surface oxygen must then recombine with another oxygen atom to form O_2_. It has been demonstrated that the addition of a reductant can facilitate the abstraction of oxygen from the oxidised active site, significantly increasing the observed rate of N_2_O decomposition at lower temperatures [27, 33–38]. Propane [26, 39–42] has been used as a reductant, in addition to ethane, methane and CO [26, 43–47].

Fe–ZSM-5 can be prepared by various ion-exchange methods, including via wet [48–51] or solid state [23, 50, 52], or sublimation [28, 32, 53–56] methodologies. Wet ion exchange includes the use of solvents, while solid state includes solventless mechanical mixing. Sublimation makes use of low evaporation temperature salts, usually FeCl_3_, as precursors. One challenge with this preparation method is that Cl^−^ ions tend to remain after sublimation and a post-preparation washing step may be required. To combat this we have used a variation of this preparation method: chemical vapour impregnation (CVI). In this method, Fe(acac)_3_ is used instead of FeCl_3_, as acetylacetonate precursors are easily removed under vacuum [57–59].

During the deposition of Fe on zeolites it is possible to form various types of Fe species such as framework Fe^3+^ (formed during isomorphous substitution), isolated Fe^3+^ or Fe^2+^ anchored to the zeolite framework by either Si–O–Fe or Al–O–Fe bridges, di-nuclear Fe–O–Fe species either in the framework or in the channels, oligomeric Fe oxo-species, and both small nanoparticles and bulk FeOx particles [26, 59–61]. Determination of the active Fe species for N_2_O decomposition remains a challenge; thus far nano-particulate iron [26, 62] and extra-framework Fe have been suggested to catalyse the decomposition of N_2_O. However, most suggest that extra framework Fe is the active species due to enabling the formation of α-oxygen, [38, 63–68] which is formed by decomposing N_2_O over reversible redox α-Fe sites that switch between Fe^2+^ and Fe^3+^ [69, 70].

Treatment of catalysts with acids was reported to increase both their activity and stability, due to the removal of spectator Fe species (Fe_x_O_y_ nano-particulates and clusters), with extended periods of time for acid washing not required to remove Fe species, with Fe being removed almost immediately [59]. Due to the stability of the zeolites, acid washing does not greatly affect the pore channels and new mesopores were not created. During mild acid washing only a small quantity of surface Al is removed [71]. This stability implies that only the Fe species present will be affected by the acid washing and the zeolite will remain unchanged [72]. Alternatively literature shows that steaming pre-treatments can be used to extract iron from the pores and into the extra-framework sites [53, 73–76], however we will not consider this technique in this work.

In this work we have investigated the importance of different Fe species in Fe–ZSM-5 for the decomposition of N_2_O in the presence and absence of a reductant, propane. In addition to comparing different Fe loadings, we have evaluated the efficacy of acid washing to increase the efficiency of the Fe in the active catalyst and we have used UV/Vis spectroscopy to identify the different Fe species.

## Experimental

### Catalyst Preparation

A series of Fe–ZSM-5 catalysts (0.4, 1.25, 2.5 wt%) were prepared by CVI following the procedure described by Forde et al. [58]. Prior to catalyst preparation, ZSM-5 (23) and (30) (Zeolyst, 2 g) were dried under vacuum, and then placed into a Schlenk flask and evacuated at room temperature using a vacuum line, followed by heating at 150 °C for 1 h under continuous vacuum to remove any surface water species. ZSM-5 (23 or 30) (Zeolyst, 0.975–0.996 g) and iron acetylacetonate Fe(acac)_3_ (Sigma Aldrich, 0.0253–0.1582 g) were placed into a glass vial and mixed by manual shaking. The obtained mixture was then transferred to a 50 mL Schlenk flask fitted with a magnetic stirrer bar and sealed. The flask was then evacuated at room temperature using a vacuum line followed by heating at 150 °C for 2 h under continuous vacuum conditions with stirring to induce sublimation and deposition of the organometallic precursor onto the support. The flask was then brought up to atmospheric pressure with air and the sample removed and calcined at 550 °C in static air for 3 h.

Acid washing was performed by heating 10 v/v% HNO_3(aq)_ (50 mL) to 50 °C, adding the catalyst (0.25 g) and stirring for 10 min. The solution was filtered and washed with deionised water (1 L g^−1^) followed by drying in an oven at 110 °C for 16 h. The samples obtained using this method were denoted as Acid Washed (AW).

### Catalyst Testing

All reactions were performed at atmospheric pressure in a continuous-flow fixed-bed reactor. A 35 cm length of 1/4 in outer diameter stainless steel tubing was packed with 0.0625 g of catalyst that was sandwiched between two layers of quartz wool. The reaction temperature was tested in the range 200–600 °C. The total flow for all reactions was 100 mL min^−1^ (GHSV of 45,000 h^−1^) and the gas feed was 5 v/v% N_2_O in He or 5 v/v% N_2_O, 5 v/v% C_3_H_8_ in He. All outgoing gaseous products were analysed online using an Agilent 7890B Gas Chromatograph (GC) [columns: Hayesep Q (80–100 mesh, 1.8 m) MolSieve 5A (80–100 mesh, 2 m)] fitted with a thermal conductivity detector.

Here we define Turnover Frequency (TOF) based on the total moles of Fe present by ICP (Eq. 2) as it is a challenge to determine the concentration of surface active sites.

2 $$Turnover\,frequency \left( {TOF} \right)\,=\,\frac{{mol\,of\,{{\text{N}}_2}{\text{O}}\,converted\,per\,second}}{{total\,mol\,of\,\text{Fe}}}$$

### Catalyst Characterisation

Diffuse reflectance UV/Vis spectra was collected using an Agilent Cary 4000 UV/Vis spectrophotometer. Samples were scanned between 200 and 800 nm (150 nm min^−1^).

X-ray photoelectron spectroscopy (XPS) was performed on a Thermo Fisher Scientific K-alpha^+^ spectrometer. Samples were analysed using a micro-focused monochromatic Al X-ray source (72 W) over an area of approximately 400 microns. Data were recorded at pass energies of 150 eV for survey scans and 40 eV for high resolution scan with 1 eV and 0.1 eV step sizes respectively. Charge neutralisation of the sample was achieved using a combination of both low energy electrons and argon ions. Data analysis was performed in CasaXPS using a Shirley type background and Scofield cross sections, with an energy dependence of -0.6.

Nitrogen adsorption isotherms were collected on a Micrometrics 3Flex. Samples (0.050 g) were degassed (250 °C, 9 h) prior to analysis. Analyses was carried out at − 196 °C with P_0_ measured continuously. Free space was measured post analysis with He. Pore size analysis was carried out using DFT (N_2_-Cylindrical Pores-Oxide surface) via the Micrometrics 3Flex software.

Inductively Coupled Plasma – Optical Emission Spectroscopy (ICP-OES) was performed by Exeter Analytical Services using HF digestion to get an accurate Fe loading. The sample was digested by Anton Paar Multiwave 3000 microwave with nitric and HF acids—then the HF was neutralised with the addition of boric acid. A reagent blank was carried out. An internal standard was added to the resulting solutions, and the blank and sample were run against Fe standards by ICP-OES using Thermo Fisher iCAP Duo 7400.

## Results and Discussion

Although iron-containing zeolites catalyse N_2_O decomposition, [24–27, 48–50, 72, 78] high reaction temperatures (> 450 °C) are typically required. The effect of varying the Si:Al ratio has been investigated previously [23, 29], the Fe:Al ratio is also an important parameter, as the maximum population of ɑ-Fe sites is directly proportional to the Al content of the zeolite [27, 50, 79]. Additionally, the presence of spectator or extraneous Fe species remains a challenge with respect to calculating real TOF values.

Fe–ZSM-5 Catalysts were prepared with Fe loadings of 0.4 and 1.25 wt% with two different Si:Al ratios. It is clear that the lower Si:Al ratio exhibits higher relative activity (Table 1).The addition of propane enhances the decomposition of N_2_O by reducing the oxidised α-Fe sites that remain on the surface of the catalysts, preventing turnover of N_2_O [27, 33–38]. Due to the higher activity of the Fe–ZSM-5 (23) parent zeolite catalyst, further investigation was carried out on this Si:Al ratio zeolite [26].

**Table 1:** Influence of Fe:Al ratio on 0.4 wt% Fe and 1.25 wt% Fe–ZSM-5 for N_2_O Decomposition both with and without propane present

Catalyst	Fe:Al ratio	N_2_O conversion at 550 °C without propane (%)	N_2_O conversion at 550 °C with propane (%)
0.4 wt% Fe–ZSM-5 (23)	0.072	20	90
0.4 wt% Fe–ZSM-5 (30)	0.092	12	81
1.25 wt% Fe–ZSM-5 (23)	0.224	35	81
1.25 wt% Fe–ZSM-5 (30)	0.288	29	68

Reaction Conditions: Total flow rate 100 mL min^−1^, 0.06 g catalyst, temperature range 400–600 °C, GHSV 45,000 h^−1^, either 5 v/v% N_2_O in He or 5 v/v% N_2_O, 5 v/v% C_3_H_8_ in He

**Fig. 1:**
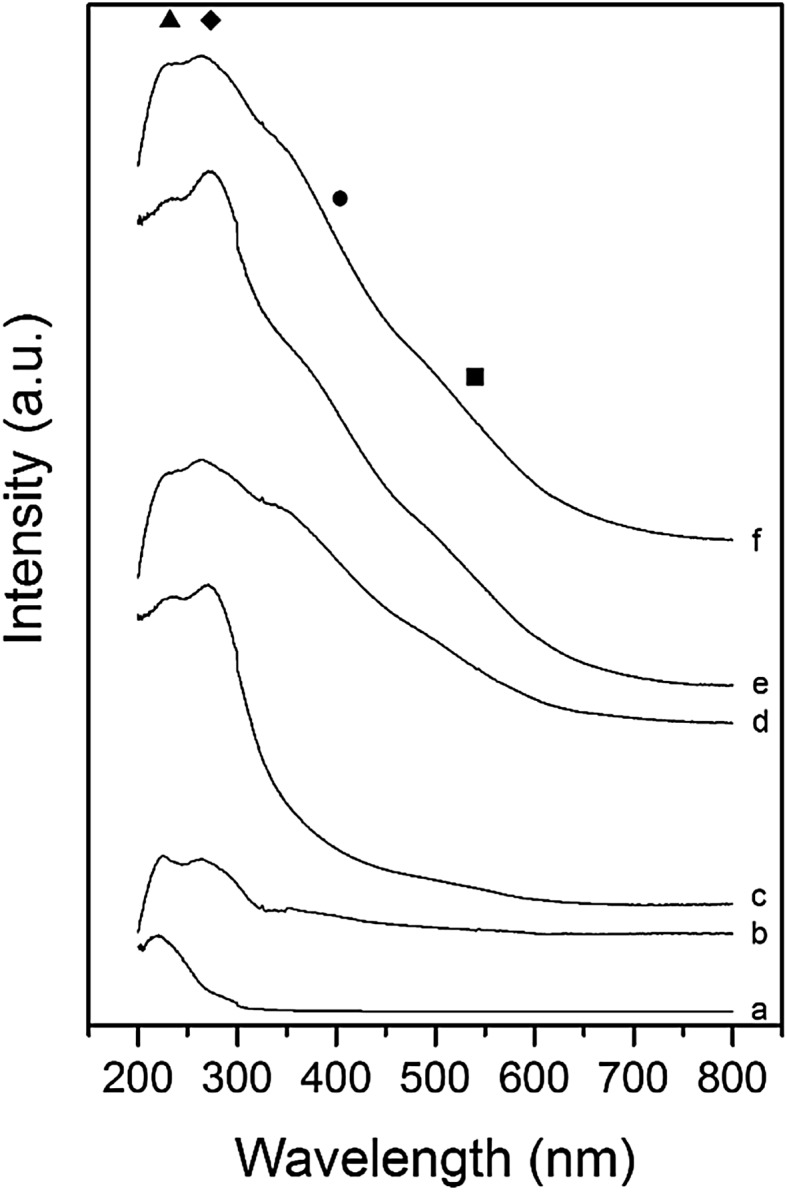
UV/Vis spectra of a series of Fe–ZSM-5 (23 or 30) catalysts and H-ZSM-5 support. *Filled triangle* framework Fe^3+^, *filled diamond* extra framework species, *filled circle* Fe_x_O_y_ clusters, *filled square* large Fe_x_O_y_ species. *a* H-ZSM-5 (23), *b* H-ZSM-5 (30), *c* 0.4 wt% Fe–ZSM-5 (23), *d* 0.4 wt% Fe–ZSM-5 (30), *e* 1.25 wt% Fe–ZSM-5 (23), *f* 1.25 wt% Fe–ZSM-5 (30)

In order to understand the effect of Si:Al ratio, UV/Vis spectroscopy of the various catalysts was performed (Fig. 1). When Fe is added to ZSM-5, four UV-active species can be differentiated. These absorb at: 200–250 nm (isolated Fe^3+^ in framework sites), 250–350 nm (isolated or oligomeric extra framework Fe species in zeolite channels), 350–450 nm (iron oxide clusters) and > 450 nm (large surface oxide species) [61, 80]. UV/Vis shows how higher Al content leads to high absorbance in the region 250–350 nm due to the presence of more extra-framework ɑ-Fe.

**Fig. 2:**
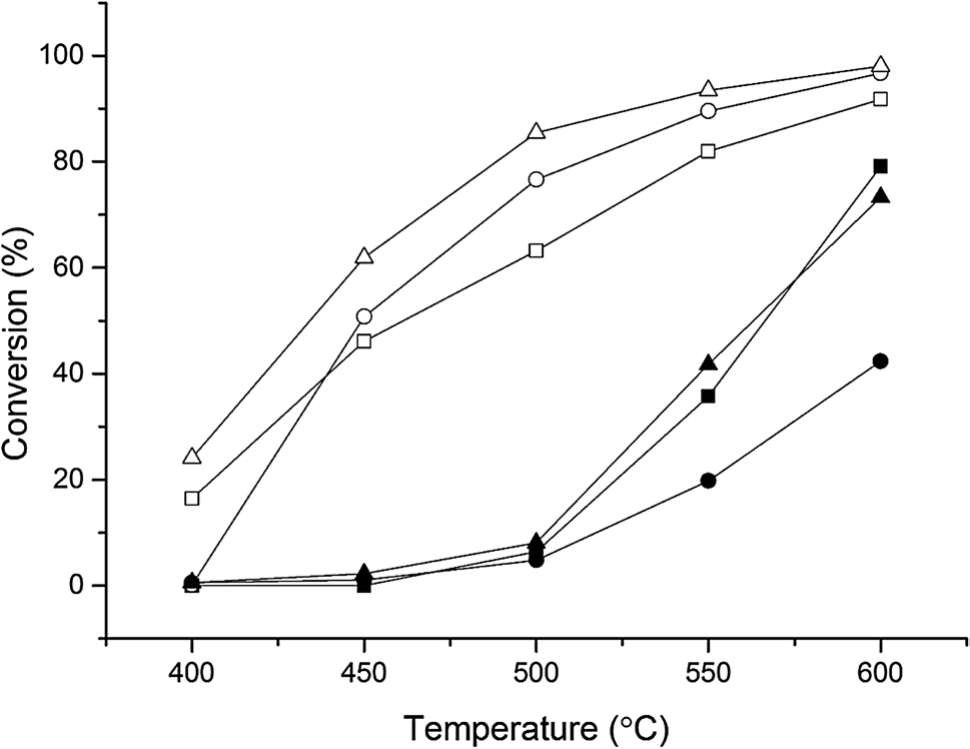
The influence of Fe weight loading on N_2_O conversion over Fe–ZSM-5 catalysts. Closed symbols: N_2_O present 5 v/v% N_2_O in He, open symbols: N_2_O + Propane present: 5 v/v% N_2_O, 5 v/v% C_3_H_8_ in He, *circle* 0.4 wt% Fe–ZSM-5 (23), *square* 1.25 wt% Fe–ZSM-5 (23), *triangle* 2.5 wt% Fe–ZSM-5 (23). Conditions; total flow rate 100 mL min^−1^, 0.06 g catalyst, temperature range 200–600 °C, GHSV 45,000 h^−1^

An additional Fe–ZSM-5 (23) catalyst was prepared with an Fe weight loading of 2.5% and contrasted to the 0.4 and 1.25 wt% catalysts. Figure 2 (closed symbols) illustrates the conversion of N_2_O over the four Fe–ZSM-5 (23) catalysts across the temperature range of 400–600 °C. The increasing weight loading of Fe in ZSM-5 increased the conversion of N_2_O, up to ca. 70% conversion over the 1.25 wt% catalyst, compared to 40% conversion over the 0.4 wt% Fe–ZSM-5 catalyst. Increasing the Fe loading to 2.5 wt% did not increase the N_2_O conversion further (Fig. 2). The 0.4% Fe catalyst exhibited limited activity despite the presence of active extra-framework ɑ-Fe species. Therefore, when N_2_O decomposition takes place at these sites oxygen recombination is limited due to the oxygen species proximity to combine to form molecular oxygen and, therefore, effectively blocking active sites.

**Fig. 3:**
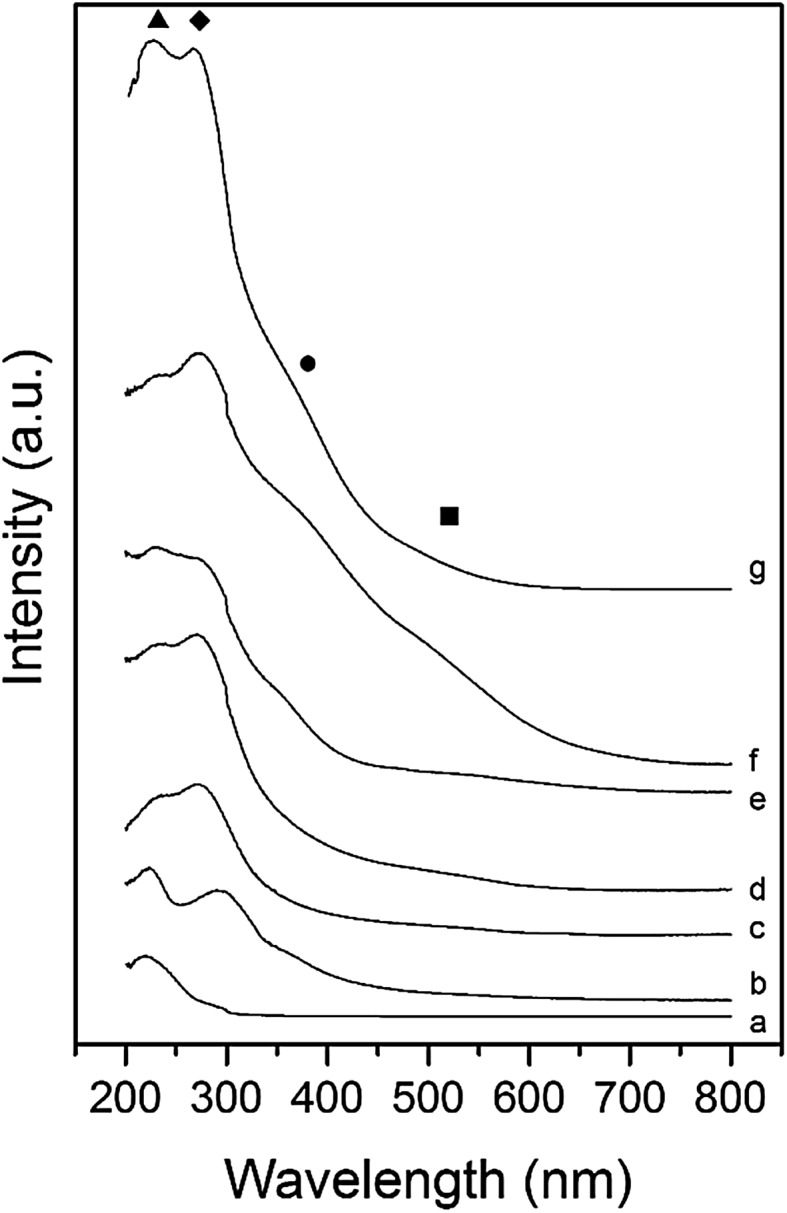
UV/Vis spectra of a series of Fe–ZSM-5 (23) catalysts and H-ZSM-5 support. *Filled circle* framework Fe^3+^, *filled diamond* extra framework species, *filled circle* Fe_x_O_y_ clusters, *filled square* Large Fe_x_O_y_ species. *a* H-ZSM-5 (23), *b* H-ZSM-5 (23) AW, *c* 0.16 wt% Fe–ZSM-5 (23), *d* 0.4 wt% Fe–ZSM-5 (23), *e* 0.4 wt% Fe–ZSM-5 (23) AW, *f* 1.25 wt% Fe–ZSM-5 (23), *g* 2.5 wt% Fe–ZSM-5 (23)

High loadings of Fe lead to a high proportion of active framework and extra-framework species and due to the increased density of these species, the rate of oxygen recombination is higher, leading to a higher conversion. UV/Vis spectroscopy (Fig. 3) shows that there are a number of distinct Fe species present in the high loaded catalysts, with both Fe_x_O_y_ nanoparticle and cluster species present, indicating that not all the Fe is efficiently utilised. Therefore, while a significant proportion of Fe is not necessarily active, there is a high concentration of extra-framework ɑ-Fe sites that can facilitate oxygen recombination and high N_2_O conversion.

When propane is added to the reaction feed-stream, the onset of activity shifts from 400 to 450 °C to a much lower temperature (Fig. 2 open symbols). In this context, propane acts as a reductant [26, 39, 40, 42, 81] and limits the formation of site blocking oxygen species on the surface of the catalyst. The rate-limiting step without propane is oxygen recombination. Propane can activate the oxidised α-Fe site forming CO and CO_2_, which regenerates the active site and allows the reaction to proceed [27, 33–36]. At lower temperatures (< 500 °C) minor quantities of propene, ethene and ethane are produced, however, at higher temperatures the selectivity shifts to exclusively CO and CO_2_.

Comparing the reaction data (Fig. 2) to the UV/Vis spectroscopy (Fig. 3), it is possible to observe that the more active catalysts have a higher proportion of framework and extra framework ɑ-Fe species. This is the case in the 0.4 wt% Fe–ZSM-5 catalyst, where UV/Vis spectroscopy (Fig. 3) shows that framework and extra-framework species are present, with only a small absorbance due to Fe_x_O_y_ nano-particles and clusters. By contrast, the poor activity of 1.25 wt% Fe–ZSM-5 correlates with the high proportion of nanoparticles and clusters of Fe_x_O_y_ present, which limit the number of Fe ions available to form the active species.

**Fig. 4:**
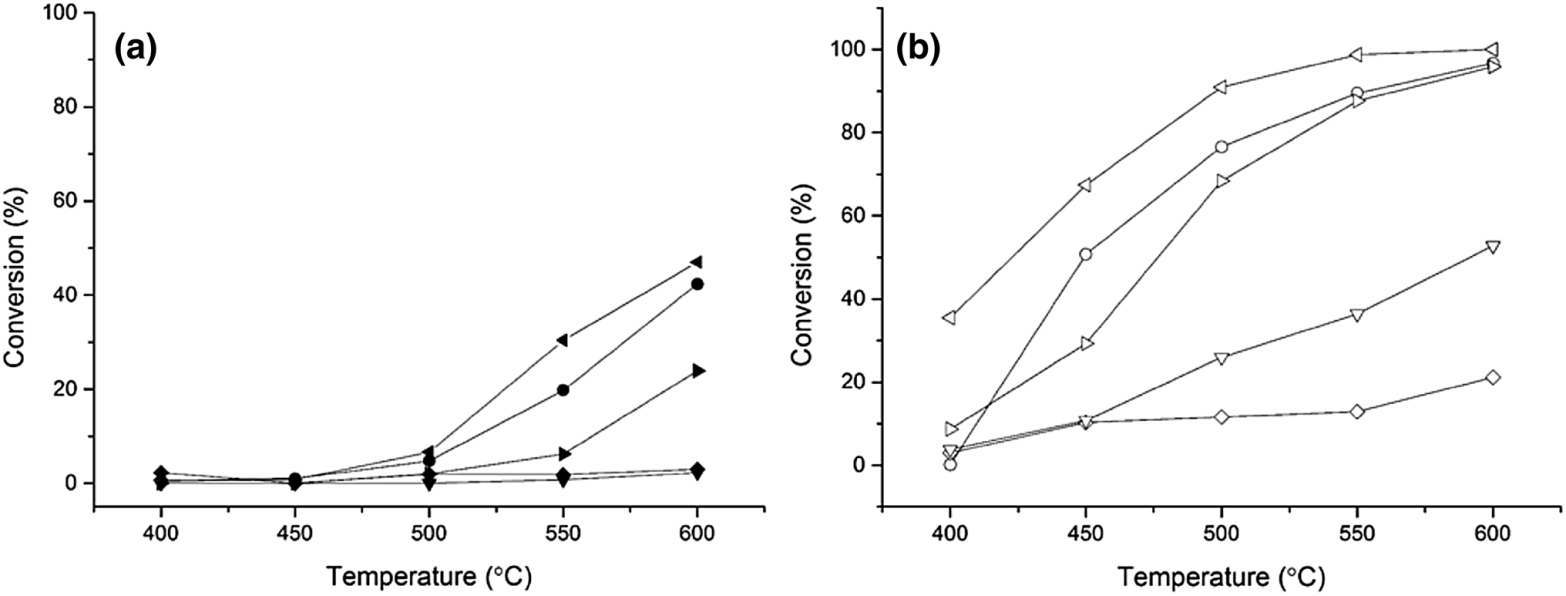
**a** Influence of Fe loading and acid washing over Fe–ZSM-5 catalysts for N_2_O conversion (closed symbols); *Left pointed triangle* 0.16 wt% Fe–ZSM-5 (23), *circle* 0.4 wt% Fe–ZSM-5 (23), *right pointed triangle* 0.4 wt% Fe–ZSM-5 (23) acid washed, *diamond* H-ZSM-5 (23), *inverted triangle* H-ZSM-5 (23) acid washed. Conditions: 5 v/v% N_2_O in He, total flow rate 100 mL min^−1^, 0.06 g catalyst, temperature range 400–600 °C, GHSV 45,000 h^−1^. **b** N_2_O conversion with propane present; open symbols. Conditions: 5 v/v% N_2_O, 5 v/v% C_3_H_8_ in He total flow rate 100 mL min^−1^, 0.06 g catalyst, temperature range 400–600 °C, GHSV 45,000 h^−1^

Peneau et al. demonstrated that using dilute HNO_3_, it is possible to remove excess iron and spectator species from the catalyst, which was investigated for the selective oxidation of ethane by H_2_O_2_ [59]. Here, 0.4 wt% Fe–ZSM-5 (23) was identified as a suitable catalyst formulation for acid washing, due to the presence of extra-framework ɑ-Fe species and minor levels of spectator Fe_x_O_y_ nano-particulates and clusters. Previous work within the group has shown that it is difficult to distinguish between the Fe species present at higher weight loadings, therefore lower weight loadings were selected for acid washing to enable changes to be noted [82, 83]. After acid washing the calcined catalyst, ICP-OES analysis of the digested samples revealed that the weight loading had reduced to 0.16%. Figure 4a illustrates the activity of the as-prepared parent catalyst, the acid washed 0.4 wt% Fe–ZSM-5 catalyst, a 0.16 wt% Fe–ZSM-5 (prepared by CVI for comparison to the AW catalyst) as well as the analogous H-ZSM-5 catalysts, which were tested to confirm that the support alone was not active for the reaction. The 0.4 wt% Fe–ZSM-5 (23) sample has an N_2_O conversion of 40% at 600 °C, however, over the acid washed catalyst the conversion was lower at 600 °C at 25%.

UV/Vis Spectroscopy (Fig. 3) supports the hypothesis that the decrease in activity observed after acid washing was due to the removal of framework Fe^3+^ ions which are extracted and deposited onto the surface of the catalyst as nanoparticles of Fe_x_O_y_ (270 nm). UV/Vis Spectroscopy of 0.16 wt% Fe–ZSM-5 suggests that the Fe is present as framework Fe^3+^ and extra-framework ɑ-Fe species only (Fig. 3). The lower activity of this catalyst further suggests that the proximity of the Fe sites to each other is crucial to achieve high activity in N_2_O decomposition. When propane is present (Fig. 4b) however, the relative proximity of active sites does not affect activity as propane can abstract oxygen from a single oxidised Fe site.

UV/Vis Spectroscopy was further used to understand the contrasting influence of the Fe loading concentration on N_2_O decomposition with and without propane. The spectrum of H-ZSM-5 (23) shows that framework Fe^3+^ species are present in the parent zeolite due to the absorbance at 220 nm (Fig. 3) and are likely to be impurities introduced during manufacture [84]. However, acid washing the H-ZSM-5 has the effect of re-dispersing the Fe species and forming Fe with extra-framework character. The 0.16 wt% Fe–ZSM-5 catalyst appears to possess both framework (< 250 nm) and extra-framework Fe (280 nm) only. Both the 1.25 wt% Fe–ZSM-5 and 2.5 wt% Fe–ZSM-5 catalysts contains all species present, with framework (< 250 nm), extra-framework Fe (280 nm), iron oxide nanoparticles (400 nm) and large clusters of iron oxide (> 450 nm). The spectrum of 0.4 wt% Fe–ZSM-5 (23) shows that there are three species of Fe present, framework Fe^3+^, extra-framework ɑ-Fe and large Fe_x_O_y_ clusters. In contrast, the spectrum of 0.4 wt% Fe–ZSM-5 (23) acid washed sample shows that there are all four species of Fe present. Most notably, a reduced absorbance due to extra- framework ɑ-Fe being extracted and an increased absorbance from deposited Fe_x_O_y_ nanoparticles and clusters.

**Fig. 5:**
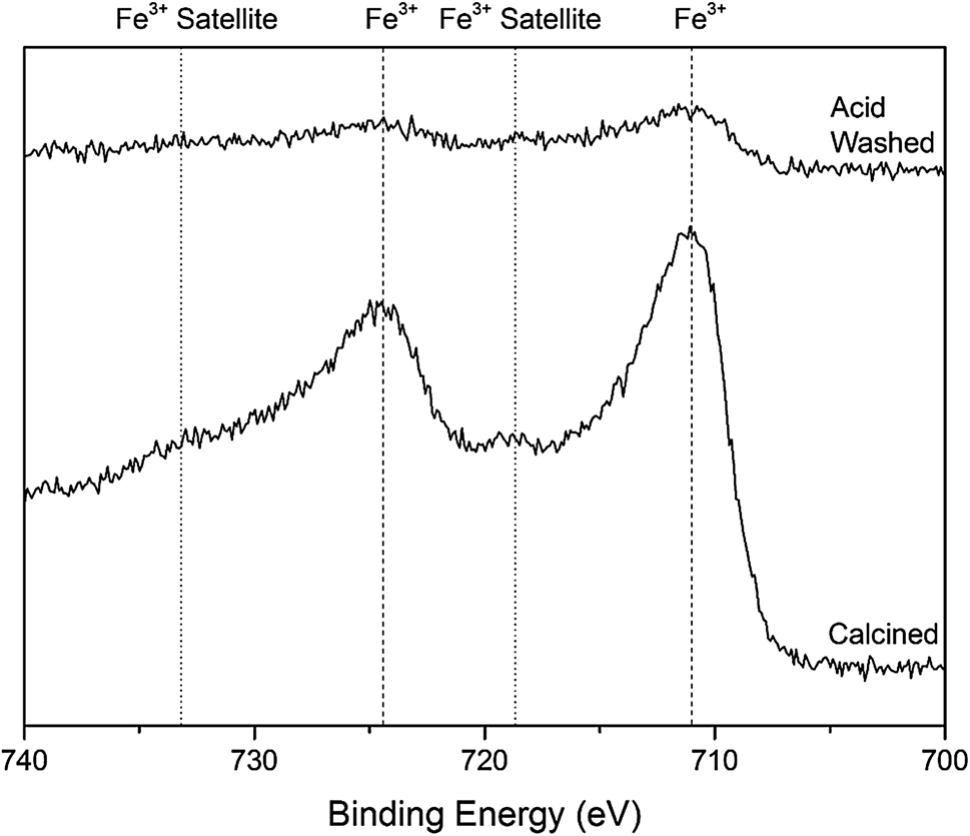
XPS data of the Fe region of 0.4 wt% Fe–ZSM-5 (23) calcined and acid washed

Further characterisation was performed on the 0.4 wt% Fe–ZSM-5 and acid washed samples with XPS (Table 2). XPS measurements revealed a significant loss of Fe from the surface of the catalyst following acid washing, as the atomic % of Fe dropped from 2.02 to 0.28%, in addition to a decrease in the intensity of the Fe peak (Fig. 5). Consideration of the surface and bulk Fe content, as determined using XPS and ICP-OES showed a drop in the surface:bulk Fe ratio after acid washing (5.05 and 1.75 for the as-prepared and acid washed 0.4 wt% Fe–ZSM-5 catalysts, respectively). This confirmed that Fe was preferentially removed from the surface of the catalyst rather than within the micro-porous channels. Furthermore, XPS showed that the binding energy of Fe is 711 eV in the calcined and acid washed catalysts, this alongside the satellite at 719 eV indicates that there is Fe^3+^ species present [85, 86]. After the addition of iron to the ZSM-5 the binding energy of both the Al and Si shift to slightly higher binding energies. Shifts in the Al spectrum from 102.9 eV in ZSM-5 to 103.4 eV in the calcined and acid washed catalyst, with Si shifting from 74.1 to 74.9 eV were observed in both catalysts. This shift to a higher binding energy indicates that Fe has substituted into the lattice, [87–89] and corresponds with the UV/Vis spectroscopy as there is a larger absorption in the framework Fe^3+^ region indicating that Fe has substituted into the framework.

**Table 2:** Surface composition, Fe binding energies, surface area and micropore volume of a series of 0.4 wt% Fe–ZSM-5 (23) catalysts and H-ZSM-5 (23) support as reported by XPS analysis. Degas conditions—9 h at 250 °C prior to analysis

Catalyst	Al 2p (at.%)	Na 1s (at.%)	O 1s (at.%)	Si 2p (at.%)	Fe 3p (at.%)	Fe binding energy (eV)	Fe satellite binding energy (eV)	Surface area (m^2^ g^−1^)	Micropore volume (cm^3^ g^−1^)
H-ZSM-5 (23)	3.17	0.41	62.52	33.90	–	–	–	423	0.167
0.4 wt% Fe–ZSM-5 (23)	3.77	0.12	49.35	44.74	2.02	711.2	719	437	0.169
0.4 wt% Fe–ZSM-5 (23) AW	3.22	0.28	49.87	46.63	0.28	711.0	719	428	0.164

Surface area measurements for all catalysts remained constant at around 430 m^2^ g^−1^. This is consistent with the parent zeolite, which has a surface area of 423 m^2^ g^−1^. The micropore volume of H-ZSM-5 (23) is 0.167 cm^3^ g^−1^, this varies by ± 0.003 cm^3^ g^−1^ when iron is added and calcined and then acid washed, (Table 2). The consistency of surface area and micropore volume during the catalyst preparation, calcination, and acid washing indicates that H-ZSM-5 (23) is stable under the pre-treatment conditions.

**Fig. 6:**
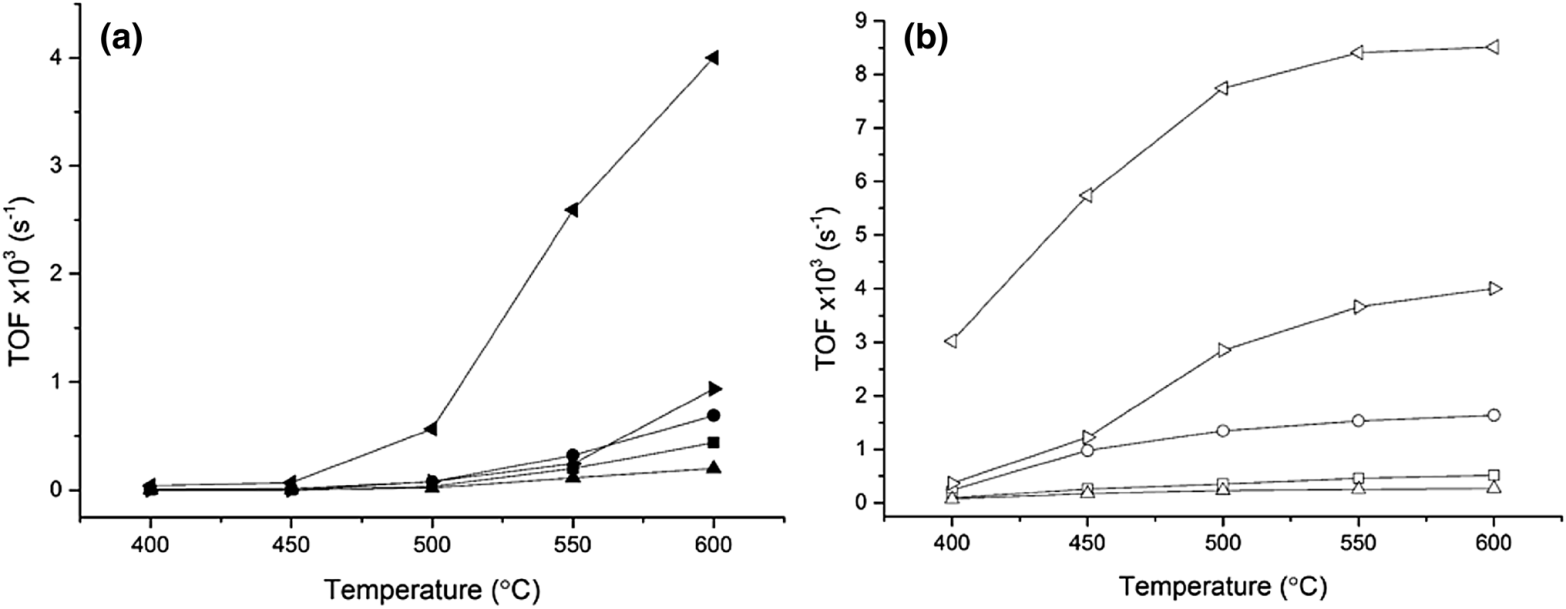
**a** TOF of N_2_O decomposition over a series of Fe–ZSM-5 catalysts that have been calcined or acid washed, closed symbols. *Left pointed triangle* 0.16 wt% Fe–ZSM-5 (23), *circle* 0.4 wt% Fe–ZSM-5 (23), *square* 1.25 wt% Fe–ZSM-5 (23), *triangle* 2.5 wt% Fe–ZSM-5 (23) *right pointed triangle* 0.4 wt% Fe–ZSM-5 (23) AW. Conditions: 5 v/v% N_2_O in He, total flow rate 100 mL min^−1^, 0.06 g catalyst, temperature range 400–600 °C, GHSV 45,000 h^−1^. **b** TOF of N_2_O decomposition with propane present, open symbols; Conditions: 5 v/v% N_2_O, 5 v/v% C_3_H_8_ in He, total flow rate 100 mL min^−1^, 0.06 g catalyst, temperature range 400–600 °C, GHSV 45,000 h^−1^

Due to the complexity in resolving the active Fe species, the TOF over the catalysts samples was calculated for N_2_O decomposition (Fig. 6a) using the total moles of Fe present in the sample. The low loaded Fe–ZSM-5 sample with 0.16 wt% Fe achieved a TOF of ca. 3.99 × 10^3^ s^−1^ at 600 °C. The TOF of the acid washed H-ZSM-5 catalyst at 600 °C is an order of magnitude greater than the Fe based catalyst, when propane is present at 600 °C (Fig. 6b). This is due to the zeolite achieving 52% conversion (Fig. 6b) and only having trace amounts of iron present (245 ppm) typically in framework positions. This results in an extremely high TOF based on the ppm of iron present, however in reality a very low yield of nitrogen was observed. The activity of the H-ZSM-5 after acid washing is due to the formation of active extra-framework ɑ-Fe from Fe that has been removed from the framework [63, 64, 90]. The TOF of the acid washed catalyst was calculated to be 0.94 × 10^3^ s^−1^, which compares to 0.69 × 10^3^ s^−1^ achieved over the parent catalyst at 600 °C (Fig. 6a). When comparing the activity of the parent and acid washed catalyst in the presence of propane, the difference in activity is less significant and at higher temperatures (> 550 °C) the activity is comparable: both catalysts achieved 95% N_2_O conversion at 600 °C (Fig. 4b). In terms of TOF, the activity of the acid washed Fe–ZSM-5 catalyst was two and a half times that of the calcined equivalent catalyst (Fig. 6b). However, the TOF over the 0.16 wt% Fe–ZSM-5 catalyst is ca. 8.5 × 10^3^ s^−1^ at > 550 °C, with propane present. Park et al. reported a TOF of 1.8 × 10^3^ s^−1^ for N_2_O decomposition at 550 °C using 1.96 wt% Fe–ZSM-5 (27) [50] compared to 2.59 × 10^3^ s^−1^ achieved by 0.16% Fe–ZSM-5 (23) at 550 °C under similar conditions, demonstrating the superior activity of the catalyst prepared herein.

## Conclusions

At low Fe loadings, Fe–ZSM-5 (23) catalysts prepared by CVI have two species of Fe present, Framework Fe^3+^ and isolated extra-framework Fe_x_O_y_ in the pores, as shown by UV/Vis spectroscopy. However, when high-loading Fe–ZSM-5 is prepared by this method there are two additional species of Fe present: Fe_x_O_y_ nanoparticles and large clusters. The species of iron present in low loaded catalysts, framework and extra-framework Fe, are the active species for N_2_O decomposition, which lead to high conversion when propane is present, however without propane the activity of these catalysts is limited by slow oxygen desorption, due to the low proximity of active Fe sites. Therefore, the oxygen desorption step becomes rate limiting. At higher weight loadings with only N_2_O present the activity of the catalyst is increased as the density of the active sites increase, therefore, increasing the rate of oxygen desorption. When acid washing is performed it is not possible to selectively remove the Fe_x_O_y_ nanoparticles and clusters, but instead extra-framework Fe is extracted from the pores and deposited on the surface, leading to a decrease in conversion, but increase in TOF.
